# Roles of Social Capital in the Association Between Internalized Homophobia and Condomless Sex Among Men Who Have Sex With Men in Southwest China: A Four-Way Decomposition

**DOI:** 10.3389/ijph.2023.1605202

**Published:** 2023-01-20

**Authors:** Bin Yu, Chuanteng Feng, Xue Yang, Zixin Wang, Huachun Zou, Peng Jia, Shujuan Yang

**Affiliations:** ^1^ Institute for Disaster Management and Reconstruction, Sichuan University-The Hong Kong Polytechnic University, Chengdu, China; ^2^ West China School of Public Health and West China Fourth Hospital, Sichuan University, Chengdu, China; ^3^ Sichuan Research Center of Sexual Sociology and Sex Education, Chengdu, China; ^4^ Jockey Club School of Public Health and Primary Care, Faculty of Medicine, The Chinese University of Hong Kong, Hong Kong, China; ^5^ School of Public Health (Shenzhen), Sun Yat-sen University, Shenzhen, China; ^6^ School of Resource and Environmental Sciences, Wuhan University, Wuhan, China; ^7^ International Institute of Spatial Lifecourse Health (ISLE), Wuhan University, Wuhan, China; ^8^ Department of Health Management Center, Clinical Medical College and Affiliated Hospital, Chengdu University, Chengdu, China

**Keywords:** social capital, men who have sex with men, internalized homophobia, condomless sex, mediation effect

## Abstract

**Objectives:** This study examined whether social capital (SC) mediated the association between internalized homophobia (IH) and condomless sex among men who have sex with men (MSM), with the interaction of SC and IH considered.

**Methods:** A cross-sectional study was conducted between November 2018 and April 2019 in Sichuan Province, China. A total of 540 participants were recruited to investigate their IH, SC, and condomless sex. A four-way decomposition of causal mediation analysis was used to test SC’s roles in the association between IH and condomless sex.

**Results:** Condomless sex was prevalent (46.7%) among the participants, which was significantly associated with IH [odds ratio (OR) = 1.70] and SC (OR = 0.55). A direct effect [excess risk ratio (RR = 0.32)] and an indirect effect (excess RR = 0.16) of SC were found to be significant in the association between IH and condomless sex. Heterogeneities in effects were observed when taking the SC’s domains (e.g., individual and family-based SC) as mediators. SC’s effects were significant only in the homosexual subgroup.

**Conclusion:** IH-based intervention with consideration of SC can be tailored to MSM to decrease condomless sex and curb the spread of HIV, especially for the homosexual subgroup.

## Introduction

Globally, HIV has become a major public health issue among men who have sex with men (MSM) [1]. It has been reported that, compared to the general male population, MSM are 28 times more likely to be affected by HIV globally [2], and this difference is about 100 times in China [3]. The overall prevalence of HIV among Chinese MSM during 2001–2018 has been estimated to be 5.7% [4]. Besides, some other sexually transmitted infections (STIs), such as the syphilis epidemic [5], also have a higher prevalence in Chinese MSM. The higher risk for STIs among MSM is mainly due to sexual risk behaviors, such as condomless sex [6]. A meta-analysis has suggested that the rate of condomless sex in Chinese MSM was 55.2% in the past 6 months [7].

Internalized homophobia (IH) is the assemblage of negative attitudes toward homosexuality in general or homosexual features and behavior among sexual minorities [8,9]. According to Meyer’s conceptualization of the Minority Stress Theory, there are distal and proximal stressors unique and chronic for minority populations. IH is a form of stigma-related proximal stressor, attributed to distal stressors (e.g., acts of antigay discrimination and victimization) [10]. Moreover, IH is in the most proximal position along the continuum from environment to self [10]. In countries with heterosexism-dominated culture, discrimination and prejudice for MSM may be pervasive [11]. Even if one’s homosexuality is concealed, MSM may be harmed by social values toward self, especially in countries where collectivist culture is highly accepted [12]. From a different perspective, symbolic interaction theorists also contend that interactions with others are important for the development of a sense of self, and negative regard from others (e.g., stigma) leads to negative self-regard (e.g., IH) [13]. Persistent IH negatively impacts health, including low self-esteem, defenses that can lead to isolation [14], anxiety, depression, and suicidal ideation [15]. Importantly, IH has further effects on risky behavior such as condomless sex [16,17] due to a low level of self-validation, coping with psychological distress resulting from the perceived stigma, and temporarily escaping from shame and depression [18,19]. Besides, a higher level of IH might be related to more substance and alcohol use that may impair decision-making processes [20]. However, the results regarding the association between IH and condomless sex remain inconsistent [21], reflecting a need for more nuanced studies, such as examining the mediator or moderator [22].

In the past 20 years, there has been a pronounced increase in studies testing potential roles of social capital in health [23]. Social capital (SC), which was described as a source of support and benefits embodied in interpersonal and social networks [24], may play an essential mediation role between mental health and risky behaviors [25–27]. The social capital theory contends that the accumulation of human capital is determined by resources of social relationships [28]. However, whether individuals can access group-level resources (e.g., medical and health services) depends on many factors, including personality and mental health [10]. For those with high IH, some group-level resources (e.g., support from family, safer sex information from MSM community) may be difficult to access. For example, due to a high level of IH, some old MSM have been cut off from their family of origin [29]. Besides, MSM indicating greater IH are more isolated from the MSM community and are less likely to discuss HIV prevention with sexual partners [30]. Therefore, IH might show a direct effect on SC, and further affect health through SC. In addition, SC has been suggested to mitigate poor mental status (e.g., stigma, and depression) and condomless sex. For example, a study conducted among 98 male sex workers in the northeast of the US has found that SC acts as a buffer in the relationship between stigma and sexual risk behaviors [16]. This has suggested that when estimating mediation effects of SC, the potential interaction effect between SC and exposure should also be considered. A qualitative study has suggested that reducing IH and fostering SC are vital elements to improve the effectiveness of HIV risk prevention in this population [31]. Although this finding is encouraging, it is unclear whether and how SC may mediate the effect of IH on condomless sex.

Moreover, individuals of stigmatized groups generally evaluate themselves by comparing with others who look similar to them [10], and MSM who are non-homosexual men have a risk of double stigma cast by gays besides heterosexuals [32]. The previous study has also suggested that MSM who are non-homosexual subgroups may be exposed to more stressors and have more mental health problems than homosexual subgroups [33]. Therefore, the association of IH, SC and condomless sex should be considered separately in these two subgroups. Besides, different SC dimensions are worth concerning due to their different roles on health outcomes [34,35].

Prior work on examining mediation effects may be limited in considering the interaction between the mediator and exposure variables. This study aimed to examine the potential mediation effect of SC on the association between IH and condomless sex in Chinese MSM, with consideration of the interaction between SC and IH. We therefore incorporated a novel causal mediation analysis with a four-way decomposition approach, which is suitable in the presence of a mediator with which the exposure may interact [36] and can help understand how much of a total effect may be attributed to mediation, interaction, both mediation and interaction, or neither. These analyses could help design targeted intervention strategies to reduce risky sexual behaviors among MSM.

## Methods

### Subjects and Recruitment

The study was conducted from November 2018 to April 2019 in the Sichuan Province of China, where the estimated HIV prevalence was the highest in China [37]. Referring to the previous report [38], we selected the participants from cities that vary in the number of MSM to reduce the potential bias in cultural diversity and social interaction in the MSM community. Based on the information provided by the Sichuan Center for Disease Control and Prevention (CDC) and the estimates of the proportion of MSM people in 2018 by the Chengdu CDC [39], we estimated the numbers of MSM people in 35 cities of Sichuan Province, which were grouped into three layers by tertiles of the number of MSM people. One city in each layer was randomly selected as a study site.

Participants were recruited through snowball sampling, and the inclusion criteria were: 1) 16 years of age or older, 2) having engaged in anal sex with males for the past 6 months, and 3) having lived in Sichuan province for the past 3 months. We trained 15 investigators from two MSM communities and six colleges, and those trained investigators then recruited potential participants online (e.g., gay dating apps such as blued, and online chat platforms) and offline (e.g., bars, teahouses, bathhouses, groves and HIV testing clinics). Specifically, for online recruitment, investigators posted the flyer on chat platforms where potential participants could chat privately with the investigators. For offline recruitment, in addition to daily testing services, investigators visited gay bars at weekends and other gay sites (e.g., teahouse) three times a week for potential participants. Those who showed interest and accepted to participate in the investigation were assured of anonymity. An electronic questionnaire was sent to online participants, and a one-on-one interview was provided by investigators to offline participants. Details of the investigation process were described in a previous study [40]. All participants recruited offline were given 30 Chinese RMB (about US $4) as compensation for their time (about 20 min for a one-on-one interview). In addition, participants recruited offline were also provided free condoms and lubricants by the investigator.

Eligible participants were screened and provided written informed consent by electronic or pen signature. A total of 817 potential participants were contacted, of whom 540 met the inclusion criteria and were analyzed. This study was approved by the ethic committee of the West China School of Public Health and the West China Fourth Hospital and was conducted in accordance with the Declaration of Helsinki (1964).

### Measurement

#### Outcome Variables

Participants reported whether they used condoms every time they had sex with a male partner for the past 6 months. The answer of “no” was considered as having condomless sex, which was used as the dependent variable.

#### Exposure Variables

The IH scale was designed according to the items of Nungesser’s inventory improved by Mayfield [8], and the IH scale of the Chinese version [41]. We modified and refined the IH scale into a 7-item scale due to the cultural differences, sensitive issues and potential response burden consideration [42]. Specifically, a panel consisting of two epidemiologists, one health psychologist, one sociologist, and four staff from gay health and culture communities participated in determining the original IH scale items. Some questions that may lead to misunderstandings due to cultural differences and some sensitive questions were screened out to obtain a 7-item scale. The scale was translated by the panel and modified in idiomatic expressions based on the feedback of the ten qualified MSM after a pre-test and panel discussion [40]. The IH scale included three dimensions: self-affirmation (one item), personal homonegativity (four items), and morality of homosexuality (two items) (Supplementary Table S1). Five-point Likert scale ranging from 1 (strongly disagree) to 5 (strongly agree) was used for all items, of which three were reverse-scored items. The IH score was measured by summing up the scores of all items, and the total score ranged from 7 to 35, with a higher score indicating a higher level of IH. The Cronbach’s *α* value of the IH scale was 0.699.

#### Mediator

The SC scale in the study was drawn from the item pool of the Chinese version of Health-related Social Capital Measurement [43,44]. The 9-item SC scale included two dimensions: individual and family (IF)-based SC (5 items) and community and society (CS)- based SC (4 items) (Supplementary Table S2). Five-point Likert scale ranging from 1 (strongly disagree) to 5 (strongly agree) was used for all items. The SC score was measured by summing up the scores of all items. The total score ranged from 9 to 45, with a higher total score indicating a higher level of SC. The Cronbach’s *α* value of the SC scale was 0.713.

#### Covariates

Demographic characteristics included age, sexual orientation, educational level, personal income, and employment status, which were considered covariates based on the knowledge of common causal precedents of both the exposure and outcome [45,46]. Besides, two HIV-related characteristics (i.e., HIV status and the number of sexual partners in the past 6 months) were also considered covariates due to their potential effects on condomless sex [47].

### Statistical Analyses

**FIGURE 1:**
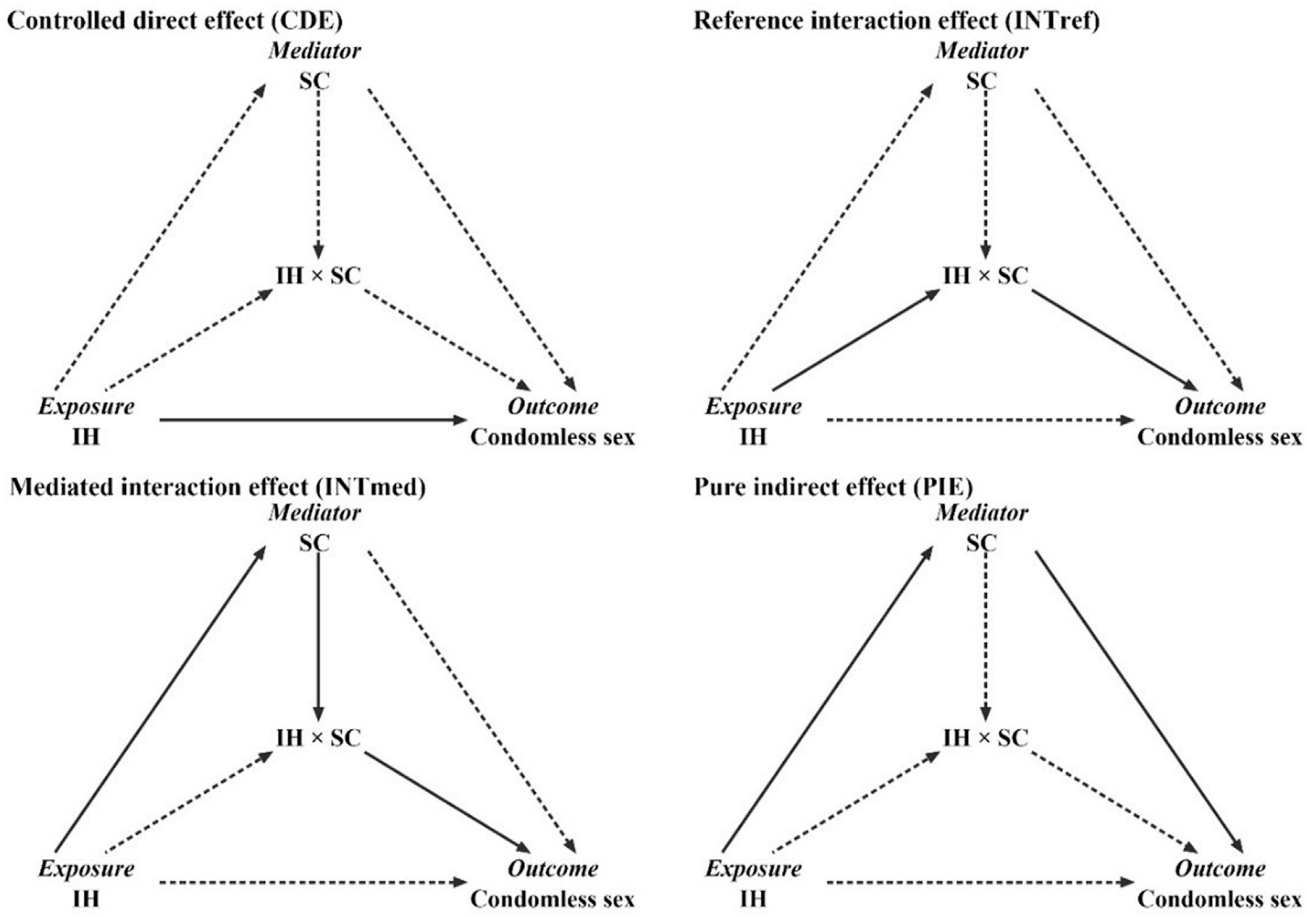
Conceptual framework of the data analysis in the current study (Chengdu, China, 2018–2019). A solid line represents a practical path, while a dashed line indicates that the path is blocked.

Descriptive statistics for categorical data (frequencies and percentages) and continuous data [median and interquartile range (IQR)] were used. Univariate and adjusted analyses were used to examine the association between IH, SC, the interaction item (IH*SC), and condomless sex. Univariate odds ratio (ORu), adjusted odds ratio (AOR), and the respective 95% confidence interval (CI) were obtained. Furthermore, the causal mediation analysis was performed using the four-way decomposition method developed by Vander Weele [36,48], which provided detailed insight into the mediation roles of SC in the association of IH and condomless sex, with total effect decomposed into four components: controlled direct effect (CDE), reference interaction effect (INTref), mediated interaction effect (INTmed), and pure indirect effect (PIE). The specific paths and definition of four-way decomposition were presented in Figure 1 and Supplementary Table S3.

The four-way decomposition method is based on a counterfactual view that fixes the exposure of all participants at a low or high level and estimates whether and how the changes in the level of mediator could affect the relationship between the exposure and outcome [36]. In this study, with SC (i.e., total SC, IF-based SC, and CS-based SC) for each participant fixed at a high level (upper half of 50th percentile for the SC score), we compared the risks for condomless sex between those with a high IH (upper half of 50th percentile for the IH score) and a low IH (lower half of 50th percentile for the IH score). Besides, we plotted the IH estimates on condomless sex due to the four components, with SC fixed across the range from the minimum to the maximum observed scores. The estimates of the total effect and four components were presented as excess risk ratio (RR), i.e., RR minus 1. Finally, subgroup analysis regarding sexual orientation (i.e., homosexual, and non-homosexual subgroups) was conducted due to the difference in condom use and IH profiles [49,50].

We used Cronbach’s *α* calculation and factor analysis (with a prerequisite of KMO > 0.6) to test the validity and reliability of the IH and SC scales used in the study. Cronbach’s *α* showed acceptable reliability for criteria of 0.6 or higher [51]. Based on Patil’s method and selection criteria [41], screen tests and parallel analysis were used to suggest components in the SC and IH scales.

Data from the offline investigation were input and derived by Epidata 3.1. Data from the online investigation were derived from the questionnaire system (https://www.wjx.cn/). R software version 4.1.2 was used for data analysis; the statistical significance was defined as *p* < 0.05.

## Results

### Descriptive Statistics

A total of 540 MSM were included in our analysis. Their median age was 31 (21, 52) years. Most of them had a college degree or above (52.4%) and a personal income of less than 2,000 Chinese yuan (41.7%). Most of them were homosexual (65.2%), and employed/students (82.6%). The percentage of condomless sex in the past 6 months was 46.7%. The median total scores of IH and SC were 17 (13, 20) and 25 (22, 29), respectively (Table 1).

**TABLE 1:** Characteristics of participants (*n* = 540) (Chengdu, China, 2018–2019).

Variables	Median (IQR) or n (%)
Condomless sex in the past 6 months	
Yes	252 (46.7)
No	288 (53.3)
IH	17 (13, 20)
SC	25 (22, 29)
Age (years)	31 (21, 52)
Homosexual	352 (65.2)
Educational level	
Middle school or below	172 (31.9)
High school/technical school	85 (15.7)
College or above	283 (52.4)
Personal income (in RMB)	
<2,000	225 (41.7)
2,000–3,999	170 (31.5)
≥4,000	145 (26.9)
Employed/student	446 (82.6)
Number of sexual partners in the past 6 months	
1	65 (12.0)
2	212 (39.3)
3	123 (22.8)
≥4	140 (25.9)
HIV-positive	37 (6.9)

Notes: IH, internalized homophobia; SC, social capital; IQR, interquartile range; RMB, “renminbi”, 1 USD = 6.99 RMB at the time of survey; STIs, sexually transmitted infections.

### Association Between IH and Condomless Sex

IH (AOR = 1.700, 95% CI [1.154, 2.504]) showed a significantly positive association with condomless sex; SC (AOR = 0.554, 95% CI [0.380, 0.807]) suggested a significantly negative association with condomless sex. The interaction of IH and SC showed a significant and positive association with condomless sex (AOR = 1.303, 95% CI [1.115, 1.522]) (Supplementary Table S4).

### Causal Mediation Analysis

**FIGURE 2:**
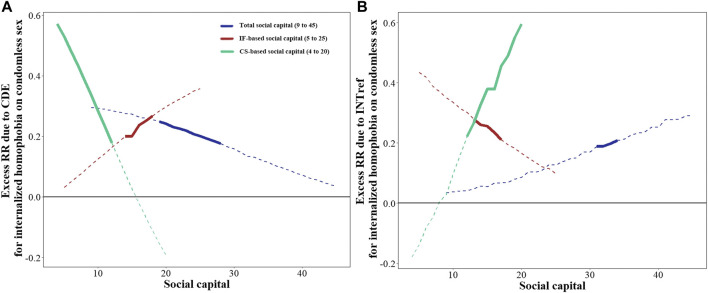
Excess risk ratio of internalized homophobia on condomless sex when social capital is fixed at a given value ranging from the minimum to the maximum observed scores in overall participants (Chengdu, China, 2018–2019). **(A)** Excess RR due to CDE when SC is fixed at a given value ranging from the minimum to the maximum observed scores. **(B)** Excess RR due to INTref when SC is fixed at a given value ranging from the minimum to the maximum observed scores. Values that range from the minimum to the maximum observed scores are listed in brackets. The solid line indicates that the excess RR is statistically significant, while the dotted line indicates that the excess RR is not statistically significant. Excess RR due to INTmed and PIE were relatively stable and therefore were not plotted (INTmed for total SC, around 0.150; for IF-based SC, around 0.003; for CS-based SC, around 0.166. PIE for total SC, around 0.156; for IF-based SC, around 0.169, for CS-based SC, around 0.039). Analyses were adjusted by age, sexual orientation, educational level, personal income, employment status, number of sexual partners in the past 6 months, and HIV status. IH, internalized homophobia; SC, social capital; IF, individual and family; CS, community and society; CDE, controlled direct effect; INTref, reference interaction effect; INTmed, mediated interaction effect; PIE, pure indirect effect; RR, risk ratio.

In overall participants, with SC as a mediator and fixed at a high level (upper half of 50th percentile for the SC score), the total effect of IH on condomless sex was significant [excess risk ratio (RR) = 0.559], and can be decomposed into four parts (Table 2). The CDE (excess RR = 0.315, *p* < 0.05), i.e., the direct effect due to neither mediation nor interaction, was significant, and there was PIE (excess RR = 0.162, *p* < 0.05), i.e., mediation effect only without interaction, in the association between IH and condomless sex. The percentages attributable of the direct effect and mediation effect only were 56.4% and 29.0%, respectively. However, the effects due to INTref (i.e., interaction effect only without mediation), and the effects due to INTmed, i.e., both mediation and interaction effect, were not significant (*p* > 0.05). When the total SC was fixed at a value from 22 to 30, the excess RRs due to direct effect significantly declined; when the total SC was fixed at a value from 29 to 34, the excess RRs due to interaction effect only significantly increased (Figure 2).

**TABLE 2:** Effects of internalized homophobia on condomless sex due to mediation and interaction with social capital in overall participants (*n* = 540) (Chengdu, China, 2018–2019).

	Total SC	%	IF-based SC	%	CS-based SC	%
	Excess RR (95% CI)		Excess RR (95% CI)		Excess RR (95% CI)	
CDE	0.315 (0.030, 0.730)**	56.361	0.365 (0.093, 0.728)***	63.468	0.183 (−0.111, 0.473)	30.541
INTref	0.144 (−0.031, 0.630)	25.792	0.201 (−0.032, 0.690)	34.916	0.321 (0.044, 0.715)**	53.690
INTmed	−0.062 (−0.415, 0.167)	−11.152	−0.080 (−0.429, 0.073)	−13.972	0.069 (−0.042, 0.291)	11.595
PIE	0.162 (0.034, 0.430)**	28.998	0.090 (−0.002, 0.366)	15.587	0.025 (−0.044, 0.133)	4.175
Total effect	0.559 (0.169, 0.334)***		0.575 (0.121, 1.246)***		0.598 (0.109, 1.195)***	

*p < 0.1, **p < 0.05, ***p < 0.01.

Analyses were adjusted by age, sexual orientation, educational level, personal income, employment status, number of sexual partners in the past 6 months, and HIV status.

%, proportion attributable; IH, internalized homophobia; SC, social capital; IF, individual and family; CS, community and society; CDE, controlled direct effect; INTref, reference interaction effect; INTmed, mediated interaction effect; PIE, pure indirect effect; RR, risk ratio.

With IF-based SC as a mediator and fixed at a high level, the excess RRs due to total effect and directed effect were 0.575 and 0.365 (all *p* < 0.01) (Table 2). With the score of IF-based SC increased, the excess RRs due to direct effect increased while due to interaction effect only showed an adverse trend (Figure 2).

With CS-based SC as a mediator and fixed at a high level, excess RRs due to total effect (excess RR = 0.598, *p* < 0.01) and interaction effect only (excess RR = 0.321, *p* < 0.05) were observed (Table 2). With the score of CS-based SC increased, the excess RRs due to interaction effect only increased while due to direct effect decreased (Figure 2).

### Subgroup Analysis

The proportion of participants with condomless sex and high IH in the homosexual subgroup was lower than that in the non-homosexual subgroup. In comparison, the proportion of participants with high SC was higher in the homosexual subgroup (Supplementary Figure S1).

**FIGURE 3:**
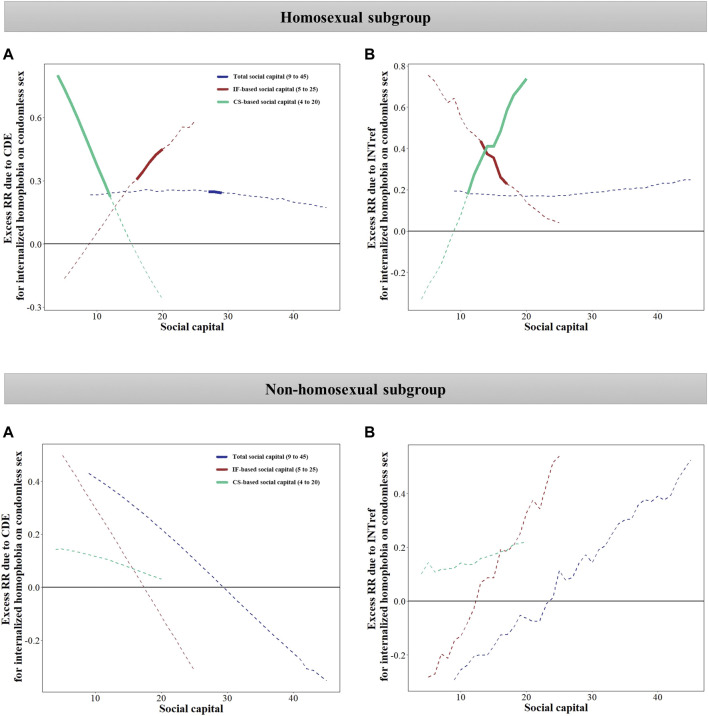
Excess risk ratio of internalized homophobia on condomless sex when social capital is fixed at a given value ranging from the minimum to the maximum observed scores in subgroups (Chengdu, China, 2018–2019). **(A)** Excess RR due to CDE when SC is fixed at a given value ranging from the minimum to the maximum observed scores. **(B)** Excess RR due to INTref when SC is fixed at a given value ranging from the minimum to the maximum observed scores. Values that range from the minimum to the maximum observed scores are listed in brackets. The solid line indicates that the excess RR is statistically significant, while the dotted line indicates that the excess RR is not statistically significant. Excess RR due to INTmed and PIE was stable and not plotted. Analyses were adjusted by age, sexual orientation, educational level, personal income, employment status, number of sexual partners in the past 6 months, and HIV status. IH, internalized homophobia; SC, social capital; IF, individual and family; CS, community and society; CDE, controlled direct effect; INTref, reference interaction effect; INTmed, mediated interaction effect; PIE, pure indirect effect; RR, risk ratio.

In the homosexual subgroup, when taking SC as a mediator and fixed at a high level, we observed significant excess RR due to total effect (excess RR = 0.854, *p* < 0.05) and mediation effect only (excess RR = 0.332, *p* < 0.05). Taking the IF-based SC or CS-based SC as mediators respectively and fixed at a high level, we found excess RR due to the total effect was significant, while heterogeneity of significant effect was observed in the directed effect and interaction effect only (Table 3). With the score of IF-based SC increased, excess RRs due to directed effect increased while excess RRs due to interaction effect only decreased; with the score of CS-based SC increased, we observed a decreased excess RR due to directed effect while an increased excess RR due to interaction effects only (Figure 3). However, in the non-homosexual subgroup, with SC or its two domains as mediators, excess RR due to four parts were all not significant at any score of SC (*p* > 0.05) (Figure 3; Table 3).

**TABLE 3:** Effects of internalized homophobia on condomless sex due to mediation and interaction with social capital in different sexual orientation groups (Chengdu, China, 2018–2019).

	Total SC	%	IF-based SC	%	CS-based SC	%
	Excess RR (95% CI)		Excess RR (95% CI)		Excess RR (95% CI)	
Homosexual (*n* = 352)						
CDE	0.329 (−0.124, 0.745)	38.480	0.615 (0.222, 1.140)***	79.186	0.224 (−0.127, 0.785)	27.921
INTref	0.192 (−0.028, 0.975)	22.487	0.325 (−0.042, 1.082)	41.934	0.437 (0.082, 1.042)**	54.575
INTmed	0.002 (−0.386, 0.481)	0.140	−0.305 (−0.890, 0.013)	−39.302	0.125 (−0.149, 0.425)	15.584
PIE	0.332 (0.073, 0.696)**	38.891	0.141 (−0.023, 0.384)	18.182	0.015 (−0.071, 0.164)	1.920
Total effect	0.854 (0.119, 1.763)**		0.776 (0.181, 1.821)**		0.801 (0.135, 1.743)**	
Non-homosexual (n = 188)						
CDE	0.200 (−0.106, 0.975)	73.436	0.059 (−0.234, 0.737)	23.035	0.059 (−0.234, 0.737)	23.035
INTref	0.058 (−0.293, 1.292)	21.492	0.133 (−0.165, 1.194)	52.140	0.133 (−0.166, 1.194)	52.140
INTmed	−0.075 (−1.056, 0.073)	−27.814	0.027 (−0.226, 0.503)	10.431	0.027 (−0.226, 0.503)	10.431
PIE	0.089 (−0.020,1.135)	32.886	0.037 (−0.084, 0.674)	14.395	0.037 (−0.084, 0.674)	14.395
Total effect	0.272 (−0.341, 1.451)		0.256 (−0.255, 1.926)		0.256 (−0.255, 1.926)	

*p < 0.1, **p < 0.05, ***p < 0.01.

Analyses were adjusted by age, sexual orientation, educational level, personal income, employment status, number of sexual partners in the past 6 months, and HIV status.

%, proportion attributable; IH, internalized homophobia; SC, social capital; IF, individual and family; CS, community and society; CDE, controlled direct effect; INTref, reference interaction effect; INTmed, mediated interaction effect; PIE, pure indirect effect; RR, risk ratio.

## Discussion

This study on Chinese MSM suggested that IH and SC were closely associated with condomless sex. Using a novel four-way composition method, we found that for those with a high level of SC, IH is still a risk factor for condomless sex. Improving SC could reduce and/or mitigate the negative effect of IH on condomless sex, with heterogeneity observed in different domains of SC. Such a mechanism was only found in the homosexual subgroup.

Social connections may have “mixed” effects and paradoxically exacerbate the effect of IH, especially if such connections entail role strain associated with obligations to provide social support to others (e.g., a man was supported and expected to carry on the family line) [52]. The heterogeneity of results in the IF-based SC and CS-based SC might be attributed to the “mixed” effects. Specifically, for CS-based SC, our study reinforced the evidence that reduction of IH could decrease condomless sex by encouraging community participation and improving government support [26,31]. In contrast to the CS-based SC, however, we observed an adverse trend for IF-based SC, i.e., the risk of condomless sex due to the direct effect of IH increased when IF-based SC was fixed at a higher level. The higher IF-based SC may be explained by the support from family members and MSM peers, which might result in more social interaction among MSM. However, for those with a high level of IH, family obligations based on strong IF-based SC become a possible stressor [52], and access to social support from other sexual minorities might be restricted [53]. They also suffer more adverse mental status (e.g., anxiety, low condom use self-efficacy) [15,54] and behavior (e.g., substance use) [20], increasing their condomless sex behavior. Besides, subgroup analysis suggested that the risk of condomless sex due to IH and the mediation and interaction effect of SC were only significant in the homosexual subgroups. However, similar to a recent study in Kazakhstan [55], our findings suggested that the non-homosexual population reported a higher level of IH, more condomless sex, and a lower level of SC than their counterpart.

We provided new findings that deserve discussion using the causal mediation analysis with the four-way method. First, with total SC fixed at a high level, mediation effects of total SC contributed significantly to the total effect, highlighting the dominant role of SC as a mediator between IH and condomless sex in MSM. Second, we found that even attained a high level of SC, MSM with a high level of IH still had a higher risk of condomless sex than those with a low level of IH. For those with a high level of SC, structural stigma may still shape the fear of being stigmatized and crisis sense of support loss from social contacts (e.g., family members, colleagues) [56,57]. External prejudice events (i.e., distal stressors) and IH might further increase the risk of condomless sex. This finding suggested that joint intervention of reducing IH and fostering SC should be designed to improve the effectiveness of HIV risk prevention [31]. Third, we found that a higher IF-based SC may not mediate or mitigate the effect of IH on condomless sex; however, risk attributable to interactions gradually increased with the improvement of CS-based SC. Those with enough community support can obtain more available health services, and self-efficacy in safe sex also mitigates the effect of IH [58].

The findings on the relationship between IH and risky sexual behavior seem mixed since the first definition of IH was proposed [59]. Williamson’s review revealed some inconsistent evidence about IH and risky sexual behavior [17]. Our study on Chinese MSM suggested a significant association of IH with condomless sex, which is in line with the findings in the US [20,30], Israel [60], and Ugandan [61]. However, inconsistencies also exist [21,62], which might be explained by one or more potential moderators [21]. Since the previous study proposed the need for more nuanced studies, such as examining the mediator or moderator [22], we, therefore, extended our previous study, which focused on the relationship between IH and sexual risk behavior [63], considering the potential role of SC in the association between IH and condomless sex. More evidence is still needed to better understand the effect of IH on condomless sex through other factors.

Our findings have strong policy implications. Although homosexuality and same-sex sexual behavior were no longer considered a disease since 2001, homosexuality is still not widely accepted [9], with structural stigma and prejudice leading to a high level of IH among Chinese MSM [64]. A previous report showed that nearly 85% of Chinese MSM kept their homosexuality secret [65]. In recent years, the Chinese government has issued some policies to enhance health service delivery for MSM [66], while mental health services for this population are still lacking [15]. As our findings suggested, those with both high SC and IH still had a high risk of condomless sex. These findings provided information on the professions or service providers of the indispensable reduction of psychological problems and minority stress like IH and to improve the family, community, and social support towards MSM to mitigate the impact of IH on condomless sex [15,26,31,67]. Further efforts are needed at civil and political levels to address structural discrimination, establish a supportive environment and strengthen peer support [68,69].

Several limitations should be mentioned in our study. First, we used a health-related SC scale for the general population and could not differentiate the SC from MSM and non-MSM peers. The estimates due to mediations and moderation of peer-related SC may differ, which requires future research. Second, the reporting bias might exist, especially for some information such as sexual orientation. However, we used anonymous questionnaires to reduce this bias as much as possible. Third, findings from the participants in Sichuan may not be generalized without caution to the overall MSM in China. Fourth, we adopted a non-probability sampling and cannot guarantee the representativeness of the total sample. Fifth, some potential covariates (e.g., pre-exposure prophylaxis use) were not included in our study, which may lead to an overestimation of the contribution of IH to the risk for condomless sex.

### Conclusions

Our study identified the mediating role of SC in the association between IH and condomless sex among Chinese MSM, and SC interacts with IH. IH is still a risk factor for condomless sex for those with a high level of SC, especially for MSM identified as homosexual. IH-based intervention with consideration of SC can be tailored to this group to decrease condomless sex and curb the spread of HIV.
