# Supplementary material for: Roles of Social Capital in the Association Between Internalized Homophobia and Condomless Sex Among Men Who Have Sex With Men in Southwest China: A Four-Way Decomposition
Source: Int J Public Health. 2023 Jan 20;68:1605202. doi: 10.3389/ijph.2023.1605202 (PMC9894890; doi:10.3389/ijph.2023.1605202)
Supplement: Supplementary file 1 [file DataSheet1.docx]

**International Journal of Public Health**

**Roles of social capital in the association between internalized homophobia and condomless sex among men who have sex with men in Southwest China: a four-way decomposition**

Table S1. Standardized factor loadings, number and proportions of participants endorsing each item on the internalized homophobia scale (Chengdu, China, 2018-2019)

| Item | Item wording (English translation) | Loading | n (%) |
| --- | --- | --- | --- |
| ***Self-affirmation*** | |  |  |
| 1 | I accept my sexual orientation | — | 458 (84.7) |
| ***Personal homonegativity*** | |  |  |
| 2 | I feel embarrassed when I think of my homosexuality | 0.824 | 120 (22.2) |
| 3 | I feel depressed when I think of my homosexuality | 0.816 | 85 (15.7) |
| 4 | I feel have deliberately alienated myself from friends and relatives sometimes for my homosexuality | 0.628 | 156 (28.9) |
| 5 | I feel afraid to reveal my homosexuality to others for fear of being looked down upon | 0.715 | 280 (51.7) |
| ***Morality of homosexuality*** | |  |  |
| 6 | In general, I believe homosexuality is as blessed as heterosexuality | 0.897 | 367 (67.9) |
| 7 | In general, I believe homosexuality is morally acceptable | 0.897 | 415 (76.7) |

n (%): number and proportion of participants that answered "agree" or "strongly agree". The loadings shown are the result of the final confirmatory factor analysis model. The loading of item 1 was not calculated due to being a single component in the initial exploratory factor analysis. The variance interpretation rate of the first principal component was 26.47%, and the cumulative variance interpretation rate was 72.32%, presenting acceptable structural validity without the common method bias.

Table S2. Standardized factor loadings, number and proportions of participants endorsing each item on the social capital scale (Chengdu, China, 2018-2019)

| Item | Item wording (English translation) | Loading | n (%) |
| --- | --- | --- | --- |
| ***Individual and family social capital*** | |  |  |
| 1 | I have many close contacts/friends | 0.817 | 164 (30.3) |
| 2 | I have many social interactions with people (e.g., your friends) in the past month | 0.702 | 161 (29.8) |
| 3 | I always trust people who have social interaction with you | 0.606 | 308 (56.4) |
| 4 | I always receive emotional/financial/instrumental support from your relatives | 0.598 | 247 (45.7) |
| 5 | I always receive emotional/financial/instrumental from your close contacts in the last year | 0.746 | 132 (24.4) |
| ***Community and society social capital*** | |  |  |
| 6 | I frequently participate in activities organized by community organizations/societies (e.g., MSM community) in the last year | 0.773 | 71 (13.1) |
| 7 | I always receive support from community organizations/societies in the last year | 0.765 | 63 (11.6) |
| 8 | I agree with the statement that hardworking people will be rewarded by the society | 0.598 | 375 (69.3) |
| 9 | I agree with the statement that talented people will be recognized by the society | 0.580 | 380 (70.3) |

n (%): number and proportion of participants that answered "agree" or "strongly agree". The loadings shown are the result of the final confirmatory factor analysis model. The variance interpretation rate of the first principal component was 32.58%, and the cumulative variance interpretation rate was 53.44%, presenting acceptable structural validity without the common method bias.

Table S3 Definitions of the components of the four-way decomposition with relevance to the current study (Chengdu, China, 2018-2019)

| Components | Counterfactual definition | Explanation | Contextual definition |
| --- | --- | --- | --- |
| Total effect (TE) | *Y_a_ − Y_a∗_* | Total effect of *A* on *Y* , with the level of *A* changing from *a^∗^* to *a* | What is the risk of condomless sex among those with high level of IH in comparison with those with low level of IH? |
| Controlled direct effect (CDE) | *Y_am_ − Y_a∗m_* | The effect of exposure *A* (changing from *a^∗^* to *a*) on the outcome *Y* ,with the mediator *M* fixed at level *m*. The CDE defines the component of TE that is due neither to interaction nor mediation | What is the risk of condomless sex among those with high level of IH in comparison with those with low level of IH, if everyone attained the same given level of SC? |
| Reference interaction (INTref ) | *(Y_am_ − Y_am∗_ − Y_a∗m_ + Y_a∗m∗_)(M_a∗_)* | An additive interaction that only operates if the mediator is present when the exposure *A* is *a*. The INTref defines the component of TE that is due to interaction only (but not mediation) | What is the combined risk of condomless sex among those with high level of IH and high level of SC, if IH does not have an effect on SC? |
| Mediated interaction (INTmed) | *(Y_am_−Y_am∗_ −Y_a∗m_+Y_a∗m∗_)(M_a_−M_a∗_)* | An additive interaction that operates if the exposure A has an effect on the mediator M (*Ma* − *Ma^∗^≠*0). The INTmed defines the component of TE that is due to both interaction and mediation | What is the combined risk of condomless sex among those with high level of IH and high level of SC, if IH has an effect on SC? |
| Pure indirect effect (PIE) | *(Y_a∗m_ − Y_a∗m∗_)(M_a_ − M_a∗_)* | The effect of the mediator on the outcome (changing from m^∗^ to m) multiplied by the effect of the exposure (changing from a^∗^ to a) on the mediator. The PIE defines the component of TE that is due to mediation only (but not interaction) | What is risk of condomless sex among those with high level of IH in comparison with those with low level of IH, if IH has an effect on SC? |

IH: internalized homophobia; SC: social capital. This table was built on the applied definitions developed by previous studies (Discacciati et al. 2018; Lai et al. 2021).

Table S4. Association of internalized homophobia, social capital, and condomless sex (Chengdu, China, 2018-2019)

|  | ORu (95%CI) | AOR (95%CI) ^a^ |
| --- | --- | --- |
| IH | 2.314 (1.635, 3.275) ^***^ | 1.700 (1.154, 2.504) ^***^ |
| SC | 0.455 (0.322, 0.642) ^***^ | 0.554 (0.380, 0.807) ^***^ |
| IH*SC | 1.462 (1.272, 1.682) ^***^ | 1.303 (1.115, 1.522) ^***^ |

^a^ adjusted by age, sexual orientation, educational level, personal income, employment status, number of sexual partners in the past six months, and HIV status.

ORu: univariate odds ratio; AOR: adjusted odds ratio; CI: confidence interval; IH: internalized homophobia; SC: social capital.

^*^P <0.1，^**^P <0.05，^***^P <0.01

**
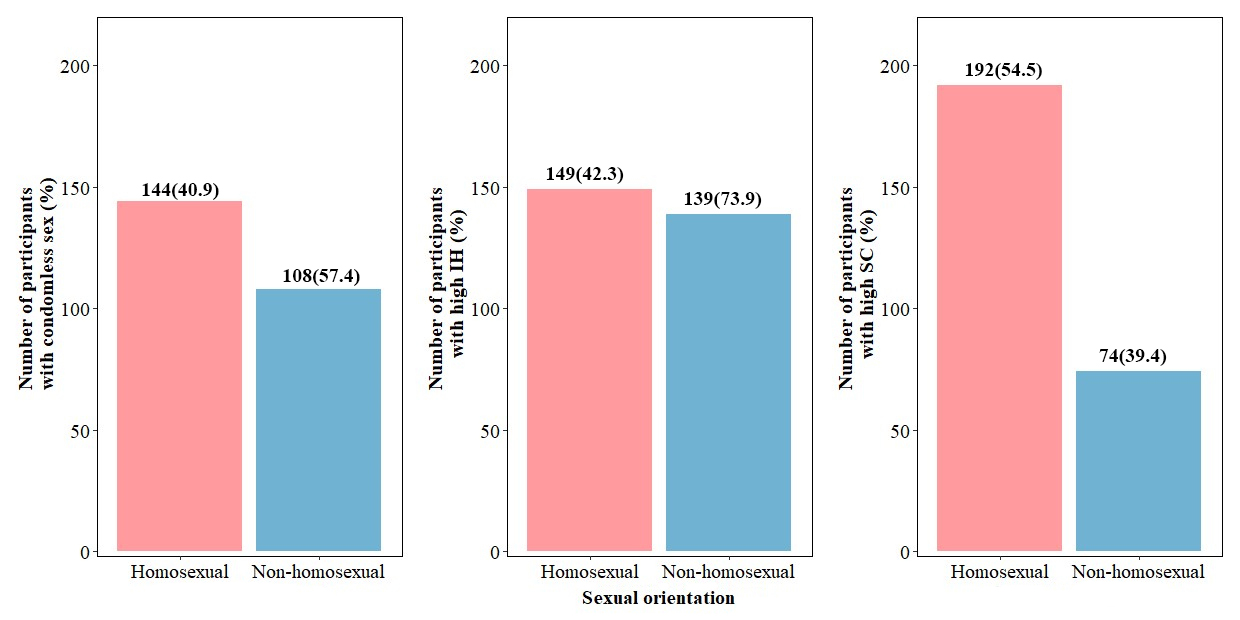
**

Figure S1. The proportion of participants with condomless sex, high internalized homophobia, and high social capital among different sexual orientations subgroups (Chengdu, China, 2018-2019)

SC: Social capital; IH: Internalized homophobia; high IH: upper half of 50th percentile for the IH total score; high SC: upper half of 50th percentile for the SC total score.

**REFERENCES**

Discacciati A, Bellavia A, Lee JJ, Mazumdar M, Valeri L (2018) Med4way: a Stata command to investigate mediating and interactive mechanisms using the four-way effect decomposition Int J Epidemiol:doi: 10.1093/ije/dyy1236. Epub ahead of print. doi:10.1093/ije/dyy236

Lai ETC, Yu R, Woo J (2021) Social gradient of self-rated health in older people-the moderating/mediating role of sense of community Age Ageing 50:1283-1289 doi:10.1093/ageing/afaa277
